# Incidence of postpartum infections and outcomes associated with antibiotic prophylaxis after normal vaginal birth

**DOI:** 10.3389/fmed.2022.939421

**Published:** 2022-09-06

**Authors:** Thitipong Sirilak, Penkarn Kanjanarat, Surapon Nochaiwong, Wasan Katip

**Affiliations:** ^1^Department of Pharmacy, Wiang Haeng Hospital, Chiang Mai, Thailand; ^2^Department of Pharmaceutical Care, Faculty of Pharmacy, Chiang Mai University, Chiang Mai, Thailand; ^3^Faculty of Pharmacy, Pharmacoepidemiology and Statistics Research Center (PESRC), Chiang Mai University, Chiang Mai, Thailand

**Keywords:** antibiotic prescription, antibiotic prophylaxis, women with vaginal delivery, rational drug use, postpartum infections

## Abstract

Antibiotic consumption accounted for approximately 15–20% of total drug costs in Thailand. From 2017 to 2018, 24.86% of Thai women who experienced vaginal delivery during normal term labour received antibiotics for postpartum infection. The Thai national practice guidelines set the target use of antibiotic prophylaxis in women following vaginal delivery of normal term labour to be no more than 10%. This study aimed to determine the incidence of postpartum infections and the outcomes and factors associated with antibiotic prophylaxis in women following vaginal delivery. The prospective cohort study was collected from 909 eligible patients who delivered infants in 7 secondary hospitals in Chiang Mai from July 2020 to February 2021. Antibiotic prescribing data and infections in women experiencing vaginal delivery during normal term labour were collected. The incidence of postpartum infections was calculated at 2 periods, 48 h and 6 weeks, after labour. Factors associated with the prescription of antibiotic prophylaxis in vaginal delivery were analysed using multivariate logistic regression. The results showed that the prevalence of antibiotic prescribing was 12.87% in a cohort of 117 patients. Postpartum infection was reported in 3 of 117 patients with antibiotics prophylaxis and 11 of 792 without antibiotics, with no statistically significant difference (RR: 1.04, 95% CI: 0.26–4.14; *p* = 0.956). Postpartum hygiene self-care practices were collected in the 6th week. The results found that there were no statistical differences in mean scores for all questions on postpartum hygiene self-care practices between the infected and non-infected groups (*p*-value > 0.05). One of the factors associated with antibiotic prophylaxis was third to fourth degree of tear and episiotomy (OR: 7.72, 95% CI: 1.13–52.75; *p* = 0.037 and OR: 2.41, 95% CI: 1.24–4.70; *p* = 0.010, respectively). There was no significance difference in postpartum infection among patients receiving antibiotic and those who did not receive antibiotics. Third to fourth degree of tear and episiotomy were significantly factors related to antibiotic prophylaxis in women with vaginal delivery after labour. This study supports practice guidelines and helps healthcare team to be assured on the use of antibiotics in no more than 10% of women experiencing normal vaginal delivery.

## Introduction

The World Health Organisation (WHO) reported that more than half of patients receive antibiotics inappropriately (1), particularly in common conditions such as common cold, acute diarrhoea, fresh wounds, and normal vaginal delivery. Particularly, antibiotics had been prescribed without indication leading to overuse of antibiotics to patients unnecessarily, and patients sometimes stop taking the antibiotics before the end of the treatment. These affect the public health system, causing an increase in antimicrobial resistance (AMR) situations (2).

Antibiotic consumption accounted for approximately 15–20% of total drug costs in Thailand (3). The Ministry of Public Health implemented strategic policy actions to alleviate, prevent and control AMR (4). A rational drug use (RDU) programme was introduced in 2013, by the FDA sub-committee promoting responsible drug use, to promote multi-sectoral collaboration to reduce antimicrobial consumption and improve public awareness; this started with antibiotic usage in upper respiratory tract infections, acute diarrhoea and fresh traumatic wounds and antibiotic prophylaxis in vaginal delivery in normal term labour (5).

From 2017 to 2018, 24.86% of Thai women experiencing vaginal delivery during normal term labour received antibiotics for postpartum infection (6). Postpartum infection is one of the leading causes of death in women after giving birth. The overall incidence of postpartum sepsis was 6%, with the incidence of sepsis following caesarean section and vaginal delivery being 7.4 and 5.5%, respectively (7). Factors associated with postpartum infection include obesity, BMI > 30, anaemia, diabetes, smoking, pregnancy over 35 years, employment, the degree of perineal wound, delivery time (more than 12 h), frequency of vaginal examination (more than 5 times) and use of equipment for delivery (8–10).

Prescribing antibiotics reduced the mortality rate and decreased the incidence of postpartum infection, in particular, surgical and vaginal deliveries with grade 3–4 perineal wounds. The study found that antibiotics in women with grade 3–4 perineal wounds were able to reduce the incidence of wound infections (RR: 0.34, 95% CI: 0.12–0.96) (11). The report from a tertiary care hospital in Thailand found that 22.7% of women received antibiotics for postpartum prophylaxis and most of antibiotics were amoxicillin (12).

The RDU programme is focused as a health service plan in Thailand (4). Practice guidelines (5) set the use of antibiotic prophylaxis in women following vaginal delivery during normal term labour to be no more than 10%. According to WHO recommendations (12) for the prevention and treatment of maternal peripartum infections, routine antibiotic prophylaxis is not recommended for women with uncomplicated vaginal births. The prevalence of antibiotic prescribing in Thailand for prophylaxis during vaginal delivery was higher than that in the RDU practice guidelines; also, there have been a limited number of studies identifying factors associated with antibiotic prescribing and the incidence of postpartum infection between antibiotics prophylaxis versus no prophylaxis in women following vaginal delivery. The aim of this study was to determine the incidence of postpartum infections and outcomes and factors associated with antibiotic prophylaxis in women following normal vaginal delivery.

## Materials and methods

### Study design and setting

This was a multi-centre prospective cohort study among women admitted for normal vaginal delivery at seven secondary care hospitals in Chiang Mai, Thailand. All women attending the study hospitals for vaginal delivery of normal term labour from July 2020 to February 2021 were recruited into the study. Women with a normal vaginal delivery, no pyrexia (body temperature < 38°C when measured in the armpits or < 38.5°C when measured in the oral cavity) and amniotic sac ruptures no later than 24 h before delivery were included in the study. Women were excluded from the study if vacuum extraction (forceps delivery) was used for delivery, antepartum or postpartum haemorrhage, and diagnosis of Group B *streptococcus* infection or the microbial cultured presence of Group B *streptococcus*. Sample size was calculated using 2-sample non-inferiority or superiority of postpartum infections during 6-week follow-up after delivery between antibiotic and non- antibiotic groups.

### Exposure of antibiotic prescribing for postpartum infection prophylaxis

Maternal intrapartum antibiotic prophylaxis (IAP) was identified as a systemic or oral antibiotic was prescribed for women with normal vaginal delivery for an indication to prevent postpartum infections and documented in hospital medical records. Antibiotic prescribing for women after delivery at discharge was also identified as antibiotic prophylaxis. Women received antibiotic for postpartum infection prophylaxis was antibiotic group. Women with no record of antibiotic prescribing for postpartum infection prophylaxis was considered a non-antibiotic or control group.

### Postpartum infections as study outcome

The criteria for determining postpartum infection were the presence of at least 2 clinical symptoms, i.e., abnormal vaginal discharge, pyrexia (oral temperature measurement more than 38.5°C), abnormal smell/foul odour discharge, delay in uterine involution (less than 2 cm per day during the first 8 days after delivery), and pelvic pain, assessed by the trained clinicians. The signs and symptoms of infection were reviewed by gynaecologists to confirmed postpartum infections at 48 h after normal delivery and at the 6-week follow-up visit.

### Data collections

The study was approved by the research ethics committee, Faculty of Pharmacy, Chiang Mai University (No. 12/2563). Written informed consent was obtained from the participants at enrolment. Trained clinicians collected data from medical records, including demographic data, data related to delivery, antibiotic prescribing, and sign and symptoms of postpartum infections at 48-h and 6-week follow-up at outpatient clinic for puerperium care. Participating clinicians were trained for participant recruitment and data collection. Factors associated with antibiotic prophylaxis were collected, including occupation, age, duration of labour, anaemia, completed antenatal care, BMI, total number of pregnancies, degree of vaginal tear. A standardised self-administered questionnaire on self-care practices and hygiene was completed by women after hospital discharge using during home stay before the 6-week follow-up. Postpartum hygiene self-care practices during 6 weeks after delivery were assessed using a 5-item self-administered questionnaire on hygienic practices including showering, handwashing, perineal hygiene, changing sanitary pad, and delaying sexual relation. A 5-point Likert scale was used to rate the practices, ranging from 1 = “never,” 2 = “rarely,” 3 = “sometimes,” 4 = “often,” and 5 = “always.” The reliability test revealed that the Cronbach’s alpha coefficient of this questionnaire was 0.73. The responses were grouped into “often-always” and “never – sometimes.”

### Data analysis

Descriptive statistics were used to describe patient characteristics. To test the differences of characteristics of women received or not received antibiotic prophylaxis for postpartum infection, student’s *t*-test was used for continuous variables and Chi-square test for categorical variables. Relative risk (RR) was calculated at 48 h and 6 weeks after delivery to indicate the incidence of postpartum infections in antibiotic and non-antibiotic groups. The study was powered to detect an 80% difference in the postpartum infection outcome. Multivariable logistic regression was performed to adjust for potential confounders, such as maternal age, history of self-administered antibiotic, length of hospital stay, duration of labour, degree of vaginal tear, anaemia, and number of pregnancies. Multivariable logistic regression was used to identify factors of antibiotic prescribing for postpartum infection prophylaxis. Statistical significance was determined by *p* < 0.05 for the study outcome. All analyses were conducted using STATA version 14.0 (STATA Corp., College Station, TX, United States).

## Results

**FIGURE 1:**
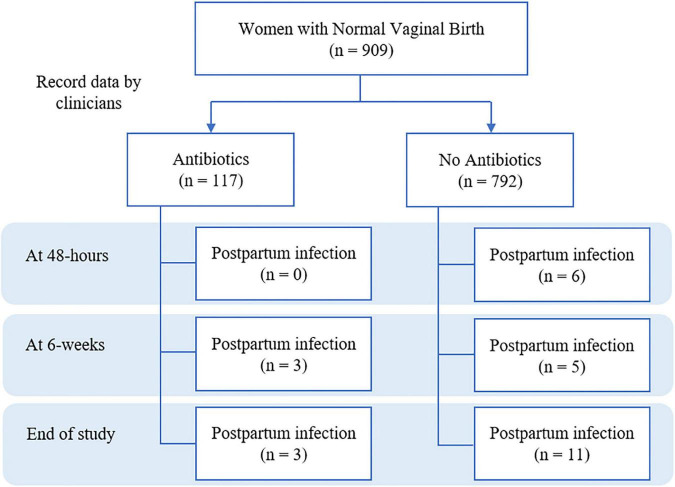
Flow chart of study on incidence of postpartum infection between antibiotics and no antibiotics prescribing.

The prospective cohort study consisted of 909 women who had a normal vaginal delivery at the secondary care hospital, Chiang Mai province, Thailand, between July 2020 and February 2021. Statistical analysis, including univariate and multivariable logistic regression were conducted to assess the incidence of postpartum infection and factors associated with antibiotic prophylaxis (Figure 1).

### Baseline characteristics

Throughout the study period, of the 909 cases, 117 women experiencing normal vaginal delivery were prescribed antibiotics for the prevention of postpartum infection, corresponding to 12.87%. Both groups were comparable in all baseline parameters, with no statistically significant difference. The majority of participants were aged between 20 and 34 years-old, with a mean age of 24.80 ± 6.47 years in the antibiotic group and 25.26 ± 6.75 years in non-antibiotic group. Most participants had a BMI ≤ 30 kg/m^2^, were unemployed and completed antenatal care 5 times (Table 1). However, patients were excluded from the study due to the presence of frequent risk factors during pregnancy or delivery for infection in the postpartum period, such as postpartum haemorrhage, for which prophylactic antibiotics are recommended. Furthermore, none of the study participants had diabetes or a smoking history.

**TABLE 1:** Baseline characteristics.

Data	Antibiotics (*n* = 117)		No antibiotics (*n* = 792)		*p*-value
		
	No.	(%)	No.	(%)	
**Hospital stay (day)**					
(Mean ± SD)	2.46	±0.93	2.20	±0.63	0.008
**Occupation**					
Yes	21	(18.0)	164	(20.7)	0.489
No	96	(82.0)	628	(79.3)	
**Age** (**year**)					
≤19	24	(20.5)	164	(21.3)	0.898
20–34	81	(69.2)	535	(69.7)	
≥35	12	(10.3)	69	(9.0)	
Average (Mean ± SD)	24.80	±6.47	25.26	±6.75	0.498
**Duration of labour**					
<12 h	86	(74.8)	597	(78.9)	0.322
≥12 h	29	(25.2)	160	(21.1)	
Median (IQR)	6.3	(10.0)	6	(7.2)	0.719
**Anaemia**					
Hb < 11 g/dl	13	(11.7)	110	(15.7)	0.282
Hb ≥ 11 g/dl	98	(88.3)	593	(84.3)	
**Antenatal care**					
Complete (5 times)	63	(57.8)	494	(64.5)	0.174
Incomplete	46	(42.2)	272	(35.5)	
**BMI (kg/m^2^)**					
≤30	110	(94.8)	694	(89.4)	0.069
>30	6	(5.2)	82	(10.6)	
**Total number of pregnancies**					
1 time	57	(49.6)	301	(38.5)	0.070
2–4 times	56	(48.7)	458	(58.6)	
≥5 times	2	(1.7)	23	(2.9)	
**Degree of vaginal tear**					
None	12	(10.2)	207	(26.1)	<0.001
First-second degree	25	(21.4)	211	(26.7)	
Third-fourth degree	2	(1.7)	4	(0.5)	
Episiotomy	78	(66.7)	370	(46.7)	

Hb: haemoglobin.

### Incidence of postpartum infection

At the end of study, 14 of the 909 cases (1.54%) were diagnosed with postpartum infection. It was found that all of them had abnormal vaginal discharge, with symptoms presenting according to other diagnostic criteria. Most of the infected women had an abnormal smell/foul odour discharge and pelvic pain. By dividing at the time of inspected at 48 h and 6 weeks, there were 6 cases and 8 cases diagnosed postpartum infection at those time, respectively.

The results on the incidence of postpartum infection between the antibiotic and non-antibiotic groups were reported. In the antibiotic group, 3 of the 117 cases had postpartum infections, while there were 11 out of 792 cases in the non-prescribed antibiotic group who had postpartum infections (Table 2). The incidence rate of postpartum infections was 18.3 per person-month in the antibiotic group and 10.0 per person-month in the non-antibiotic group, respectively.

**TABLE 2:** Incidence rates of postpartum infections by antibiotic prophylaxis status.

Outcome parameter	No. of patients (%)		Unadjusted risk ratio (95% CI)	*P* value	Adjusted * risk ratio (95% CI)	*P* value
	
	Antibiotic (*n* = 117)	Non-antibiotic (*n* = 792)				
Postpartum infection	3 (2.6)	11 (1.4)	1.85 (0.52–6.52)	0.341	1.04 (0.26–4.14)	0.956
**Postpartum infection by degree of vaginal tear**						
None	0	0	NA		NA	
First-Second degree	2 (1.7)	1 (0.1)	0.52 (0.14–1.84)	0.308	0.67 (0.17–2.57)	0.563
Third-fourth degree	0	0	NA		NA	
Episiotomy	1 (0.9)	10 (1.3)	NA		NA	

*Adjusted for covariates: antibiotic prophylaxis, age, hospital stays, duration of labour, degree of vaginal tear, and total number of pregnancies.

The most commonly prescribed antibiotic in the hospital for prophylactic infection in 117 cases was oral amoxicillin, with a usage level of 77 times in 63 cases, as a percentage 53.8% in the antibiotic group. The next highest were ampicillin and ceftriaxone in 31 cases (26.5%) and 14 cases (12%), respectively.

### Factors associated with antibiotic prophylaxis

Univariable and multivariable logistic regression were used to determine factors associated with antibiotic prescription for prophylaxis postpartum infection in women following normal vaginal delivery. The results showed that the degree of tear as third to fourth degree and episiotomy were a statistically significant factor for antibiotic prescribing in women experiencing normal vaginal delivery (OR: 7.72, 95% CI: 1.13–52.75; *p* = 0.037 and OR: 2.41, 95% CI: 1.24–4.70; *p* = 0.010, respectively; Table 3).

**TABLE 3:** Factors associated with antibiotic prescribing for postpartum infection prophylaxis among women with normal vaginal delivery.

Factors	Unadjusted OR (95% CI)	*p*-value	Adjusted OR* (95% CI)	*p*-value
Occupation	0.84 (0.51–1.38)	0.490	0.86 (0.49–1.52)	0.616
Age ≥ 35 years	1.16 (0.61–2.21)	0.657	1.52 (0.71–3.26)	0.286
Duration of labour > 12 h	1.26 (0.80–1.98)	0.323	1.27 (0.76–2.12)	0.357
Anaemia (Hb < 11 g/dl)	1.40 (0.76–2.58)	0.284	1.43 (0.75–2.76)	0.280
Completed antenatal care (≥5 times)	0.75 (0.50–1.13)	0.175	0.66 (0.42–1.02)	0.064
BMI > 30 (kg/m^2^)	0.46 (0.20–1.08)	0.076	0.52 (0.21–1.25)	0.145
**Total number of pregnancies**				
1 time	1.00		1.00	
2–4 times	0.64 (0.43–0.96)	0.031	0.66 (0.42–1.05)	0.080
≥5 times	0.46 (0.10–2.00)	0.300	0.66 (0.14–3.25)	0.614
**Degree of vaginal tear**				
None	1.00		1.00	
First-second degree	2.04 (1.00–4.18)	0.050	1.71 (0.80–3.670)	0.165
Third-fourth degree	8.62 (1.43–51.88)	0.019	7.72 (1.13–52.75)	0.037
Episiotomy	3.64 (1.93–6.84)	<0.001	2.41 (1.24–4.70)	0.010

Hb: haemoglobin. *Adjusted for covariates: antibiotic prophylaxis, age, hospital stays, duration of labour, degree of vaginal tear, and total number of pregnancies.

### Postpartum hygiene self-care practices among postpartum infection and non-infection groups

From a total of 909, there were 428 women responded to the questionnaire, 5 women in the infection group and 423 women in the non-infection group. The results indicated that there were no statistical differences of the proportions of postpartum self-care practices and the mean scores of all items between the infected and non-infected groups (*p*-value > 0.05; Table 4).

**TABLE 4:** Postpartum hygiene self-care practices in infected and non-infected groups.

Item	Postpartum hygiene self-care practices	Infection (*n* = 5)		Non-infection (*n* = 423)		*p*-value
			
		No.	(%)	No.	(%)	
**1**	**Take a shower at least twice a day**					
	Often – Always	1	(20.0)	219	(51.8)	0.204*
	Never – Sometimes	4	(80.0)	204	(48.2)	
	Average score (Mean ± SD)	3.20	±1.10	3.47	±0.97	0.543**
**2**	**Wash hands with soap and water regularly or often use hand sanitizer after touching objects and surfaces, using the toilet, or changing diaper.**					
	Often – Always	3	(60.0)	283	(66.9)	0.668*
	Never – Sometimes	2	(40.0)	140	(33.1)	
	Average score (Mean ± SD)	4.20	±1.10	3.85	±0.84	0.352**
**3**	**Cleaning perineum after urination by wiping from front to back**					
	Often – Always	3	(60.0)	305	(72.1)	0.623*
	Never – Sometimes	2	(40.0)	118	(27.9)	
	Average score (Mean ± SD)	4.20	±1.10	3.95	±0.78	0.480**
**‘**4	**Changing sanitary pad when soiled or every 3–4 h by pulling the pad from front to back**					
	Often – Always	3	(60.0)	334	(79.0)	0.288*
	Never – Sometimes	2	(40.0)	89	(21.0)	
	Average score (Mean ± SD)	4.00	±1.00	4.14	±0.78	0.682**
**5**	**Avoiding sexual relations for 4–6 weeks after childbirth**					
	Often – Always	5	(100.0)	387	(91.5)	N/A
	Never – Sometimes	0	(0.0)	36	(8.5)	
	Average score (Median, IQR)	5.00	0	5.00	1	0.131**
	Total	20.6	±3.50	20.0	±2.93	0.643

*Chi-squared test. **Student’s t-test.

## Discussion

This study found that 117 of a total 909 women with normal vaginal delivery (12.87%) were prescribed antibiotics for postpartum infection prophylaxis, of which 53.8% of the women received oral amoxicillin. Postpartum infection was found in 3 of 117 women treated with antibiotics and 11 of 792 women with no antibiotic prophylaxis (OR: 1.04, 95% CI: 0.26–4.14; *p* = 0.956). At the six-week follow-up visit, the mean scores of postpartum hygiene and self-care were not different between women received antibiotics and no antibiotics (*p*-value > 0.05). The level of tear in third to fourth degree (OR: 7.72, 95% CI: 1.13–52.75; *p* = 0.037) and episiotomy (OR: 2.41, 95% CI: 1.24–4.70; *p* = 0.010) were the factors associated with antibiotic prescribing for postpartum infection prophylaxis.

An Indian study (13) found that antibiotics were prescribed in as many as 87% of women. The majority of antibiotics prescribed (35%), were third generation cephalosporins. The study at a tertiary care hospital in Thailand (12) reported that 22% of patients were prescribed antibiotics for prevent postpartum infections, and the most commonly used antibiotic was oral amoxicillin (93.62% of cases). It is found that the antibiotic prescribing rate in India is much higher than Thailand. This might be due to the public health problem in the country, with a high rate of maternal infection and which leads to mortality in India. Thus, antibiotic treatment aims to reduce the rate of infection. However, antibiotic prescription in Thailand is still higher than recommendations from the Thai RDU hospital programme which recommended antibiotic prescription of no more than 10% in women with normal vaginal delivery. Considering the prescription of antibiotics in Thailand, oral amoxicillin is not suitable for use in the prevention of postpartum infections because it is ineffective on the most common organisms in postpartum infection, including *Staphylococcus aureus*, *Escherichia coli*, group B *streptococci* and anaerobes, such as *Bacteroides* spp. (14, 15). The rational drug use guidelines of Thailand (4) and WHO guidelines (1) recommend the use of single intravenous antibiotics, 1–2 g of cefazolin or 3 g of ampicillin-sulbactam, within 60 min prior to suturing in cases of third to fourth degree tears. In the case of hypersensitivity to penicillin, 600–900 mg of clindamycin is recommended. This is similar to the recommendations of the American College of Obstetricians and Gynaecologists (16) which recommends the use of first-generation cephalosporin or clindamycin for penicillin allergy. As a result, only 1.7% of antibiotic prescriptions use a single injection of cefazolin and this seemed to be an inappropriate use of antibiotic prescriptions for postpartum prophylaxis in Thailand.

The incidence of postpartum infections in women with normal vaginal delivery was followed-up at 48 h and 6 weeks after delivery by trained nurses. It was found that 1.54%, or 14 cases out of the 909 cases, were diagnosed with postpartum infections in this study. The study by Yokoe et al. (7) from the United States reported overall rates of infection after childbirth and vaginal delivery of 6 and 5.5%, respectively. This might be the postpartum infection diagnosis and group designation which included other in-hospital diagnoses, including emergency rooms and outpatient departments, such as mastitis, urinary tract infections, urinary tract infections, surgical site infection, endometritis and perineal wound infection. More than 80% of them had mastitis and urinary tract infections. However, the study did not reveal the number of pregnant women who received antibiotics and types of antibiotics used to prevent infections. Another study in tertiary care hospitals Thailand at Mahasarakham Hospital (17) used retrospective data between October 1 2015 and April 30 2018 and found that 2 out of 3,660 women reported postpartum infection after normal vaginal delivery, representing a rate of 0.05%. Since infected individuals might go to another hospital, preventing data collection from medical records when patients returned for follow-up after delivery, the infection rate was lower than usual. Moreover, Mahasarakham Hospital is a tertiary care hospital with a difference from this research study, with eight secondary care hospitals, as there was an obstetrician and gynaecologist who performed the delivery in this population of pregnant women. When considering the antibiotics prescribed, the tertiary care study prescribed oral amoxicillin in about 51.1% of cases, similar to this multi-research site study (17).

When analysing data of postpartum infections, it was shown that 3 of the 117 cases (or 2.6%) in the antibiotic-treated group were infected compared to the non-antibiotic groups, with 11 out of 792 cases (1.4%). There was no statistically significant difference in the proportion of postpartum infections between the two groups (*p* = 0.956). The systematic review by Bonet (23) on the use of antibiotics to prevent infection in women after delivery found that the risk of urinary tract infections, wound infections and length of hospital stay in women treated with antibiotics were not different when compared to those who received placebo or non-antibiotics. In the study by Tandon and Dalal (18), the symptoms of infection were 0.7 and 2% in the antibiotic-treated group and the untreated group, respectively. There was no statistically significant difference in symptoms of infection in either group (*p* < 0.622). Another study in Thailand (19) compared complication outcomes in the antibiotic-treated group versus the no antibiotic group and found that neither group had complications in postpartum sepsis. Postpartum infections, between 57 women treated with amoxicillin compared to 56 women without amoxicillin, were not found in either group (20). The infection assessment criteria were similar in this study. The results of these studies showed that the outcome of postpartum infection was not different between antibiotic and non-antibiotic use in women with normal delivery. Therefore, antibiotic prophylaxis should not be given to prevent postpartum infections in women who have a normal vaginal delivery, except in patients with third to fourth degree tears. The WHO (1) and Thailand’s Rational prescribing guidelines (5) recommended a single intravenous injection antibiotic within 60 min before suturing with lists of antibiotics, including cefazolin 1–2 g or ampicillin-sulbactam 3 g and clindamycin 600–900 mg, for patients who show hypersensitivity to penicillin. This was ineffective against the causative organisms of postpartum infections, including *Staphylococcus aureus*, *Escherichia coli* and *Streptococcus* species and anaerobic bacteria such as *Bacteroides* spp. Based on the findings of this study, two of the six cases of third to fourth degree tears were treated with oral amoxycillin, which abused the guidelines for antibiotic use in the prevention of postpartum infections. Additionally, there are no culture-confirmed bacterial pathogens causing infections in diagnosed cases with postpartum infections, which is a study limitation because these infections are likely under-reported in inpatient charts.

A study of factors associated with antibiotic prescription to prevent infection in women after normal vaginal delivery found that the factors associated with antibiotic prescription by doctors included the degree of tear, especially the level third to fourth degree of tear and episiotomy (*p*-value = 0.037 and *p*-value = 0.010). According to the study by Sharma et al. (21), 81.1% of women with perineal episiotomy were prescribed antibiotics. The most commonly prescribed antibiotics in the 5-day oral form were ampicillin, amoxicillin and cephalexin. The study by Bonet (24) reported that the use of antibiotics in episiotomy was not statistically different in infected wounds between the antibiotic-treated or non-antibiotic groups. WHO guidelines (1) do not recommend the prescription of antibiotics for the empirical treatment of postpartum infection in women with episiotomy lesions. Also, the American College of Obstetricians and Gynaecologists (22) does not recommend the use of antibiotics in the prevention of postpartum infection.

## Conclusion

At the end of the study, 14 out of 909 cases (1.54%) were diagnosed with postpartum infection. The incidence of postpartum infection was 1.04 times (3/114 women) higher in the antibiotic group compared to the non-antibiotic group (11/781 women). There was no significance difference in the use of antibiotics or not for prophylaxis in postpartum infection. The degree of tear at the level of third to fourth degree and episiotomy are the factors considered when prescribing antibiotics to prevent infection in women following vaginal delivery. Evidence-based practice using data to empower and encourage healthcare teams in the awareness of prescribing antibiotics to no more than 10% of patients following vaginal delivery in normal term labour for the prevention of postpartum infection.

## Data availability statement

The raw data supporting the conclusions of this article will be made available by the authors, without undue reservation.

## Ethics statement

The studies involving human participants were reviewed and approved by ethics committee on human research of the Faculty of Pharmacy, Chiang Mai University (12/2563). The patients/participants provided their written informed consent to participate in this study.

## Author contributions

TS, PK, and WK: conceptualization, methodology, writing – original draft, and writing – review and editing. TS: data curation, formal analysis, investigation, project administration, and software. PK and WK: supervision. SN: validation. All authors contributed to the article and approved the submitted version.
